# Supplementary material for: Binding of Akkermansia muciniphila to mucin is O-glycan specific
Source: Nat Commun. 2024 May 29;15:4582. doi: 10.1038/s41467-024-48770-8 (PMC11137150; doi:10.1038/s41467-024-48770-8)
Supplement: Supplementary file 1 — Supplementary Information [file 41467_2024_48770_MOESM1_ESM.pdf]

Supplementary Information

Elzinga *et al.*

Binding of *Akkermansia muciniphila* to mucin is O-glycan specific

Janneke Elzinga[1,2], Yoshiki Narimatsu[2,3], Noortje de Haan[2,5], Henrik Clausen[2], Willem M. de Vos[1,4,*], Hanne L.P. Tytgat[1,6,*]

[1] Laboratory of Microbiology, Wageningen University & Research, Wageningen, The Netherlands

[2] Copenhagen Center for Glycomics, Department of Cellular and Molecular Medicine, Faculty of Health Sciences, University of Copenhagen, Copenhagen, Denmark

[3] GlycoDisplay ApS, Copenhagen, Denmark

[4] Human Microbiome Research Program, Faculty of Medicine, University of Helsinki, Helsinki, Finland

[5] Current address: Center for Proteomics and Metabolomics, Leiden University Medical Center, Leiden, The Netherlands

[6] Current address: Nestlé Institute of Health Sciences, Nestlé Research, Lausanne, Switzerland

*These authors jointly supervised this work

Supplementary Figure 1: ELISA binding assays of *Akkermansia muciniphila* to porcine gastric mucin (PGM).

Supplementary Figure 2: Quantification of mucin TR reporters.

Supplementary Figure 3: nanoLC-MS analysis of released O-glycans from MUC2 WT and core3 mucin TR reporters.

Supplementary Figure 4: Flow cytometry analysis of mucinase activity of pasteurized *A. muciniphila*.

Supplementary Figure 5: Flow cytometry analysis of expression of mucin TR reporters in glycoengineered HEK293 cells.

Supplementary Figure 6: ELISA binding assays of live *Akkermansia muciniphila* to mucin TR reporters.

Supplementary Figure 7: Endogenous neuraminidase activity of *A. muciniphila*.

Supplementary Figure 8: Comparison of binding between growth phases.

Supplementary Figure 9: Quantification of activity of *C. perfringens* and *A. muciniphila* sialidases using MU-NANA assay.

Uncropped scan of gel presented in Supplementary Figure 2

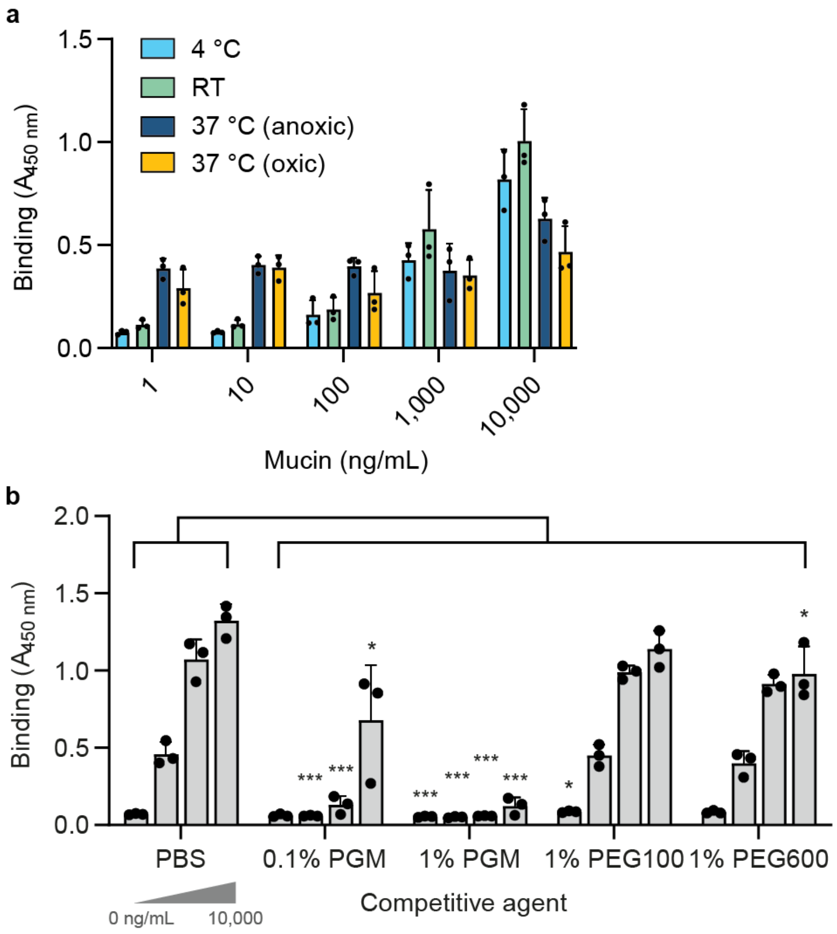

Supplementary Figure 1: ELISA binding assays of *Akkermansia muciniphila* to porcine gastric mucin (PGM). a Binding of pasteurized *A. muciniphila* ($5 \cdot 10^8$ CFU/mL in PBS) under oxic conditions at different temperature*s* (4 °C, RT or 37 °C) and under anoxic conditions at 37 °C to varying concentrations of PGM. **b** Inhibition of binding of live *A. muciniphila* ($5 \cdot 10^8$ CFU/mL) with polymers PEG (100 and 600 kDa) or PGM at 4 °C under oxic conditions. Bars and data points represent the mean ± SD of three biological replicates. Source data are provided as a Source Data file.

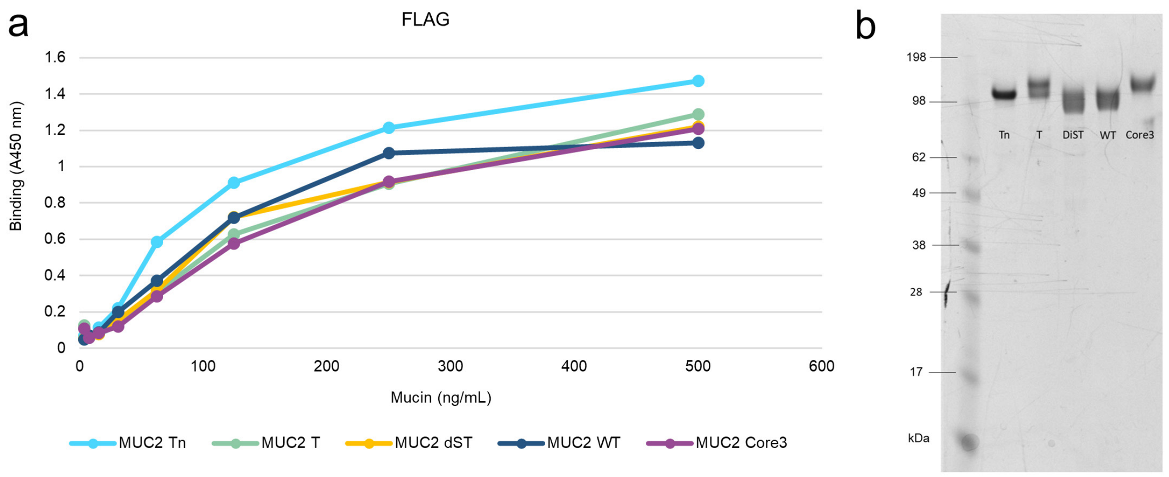

Supplementary Figure 2: Quantification of mucin TR reporters a ELISA assays of purified mucin TR reporters coated at varying concentrations and detected with anti-FLAG antibody (0.1 µg/mL). Data points represent the mean of 2 technical replicates. **b** SDS-PAGE Coomassie blue analysis of purified MUC2 reporters (1 µg loaded). This experiment was performed once. Source data are provided as a Source Data file.

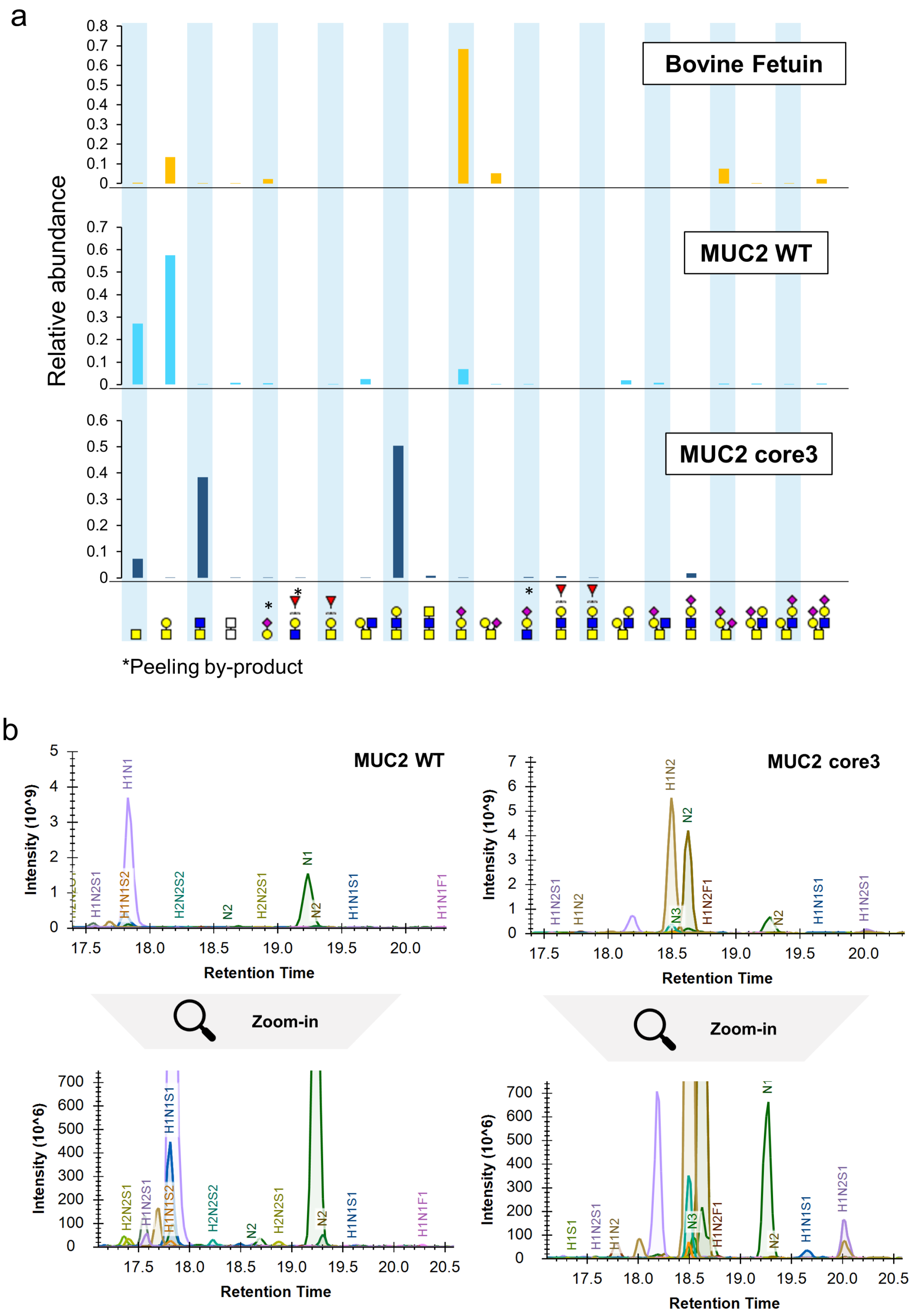

Supplementary Figure 3: nanoLC-MS analysis of released O-glycans from MUC2 WT and core3 mucin TR reporters. a Relative abundance of identified O-glycans. Fetuin was included as control for peeling (< 10%) and sialic acid loss (no indications). Bars represent n = 1. **b** Retention times for the various glycans measured labeled by their monosaccharide composition, where H = Hexose, N = *N*-Acetylhexosamine, F = Fucose and S = *N*-Acetylneuraminic acid. Unlabeled signals belong to O-glycan structures other than GalNAc-type O-glycans. Source data are provided as a Source Data file.

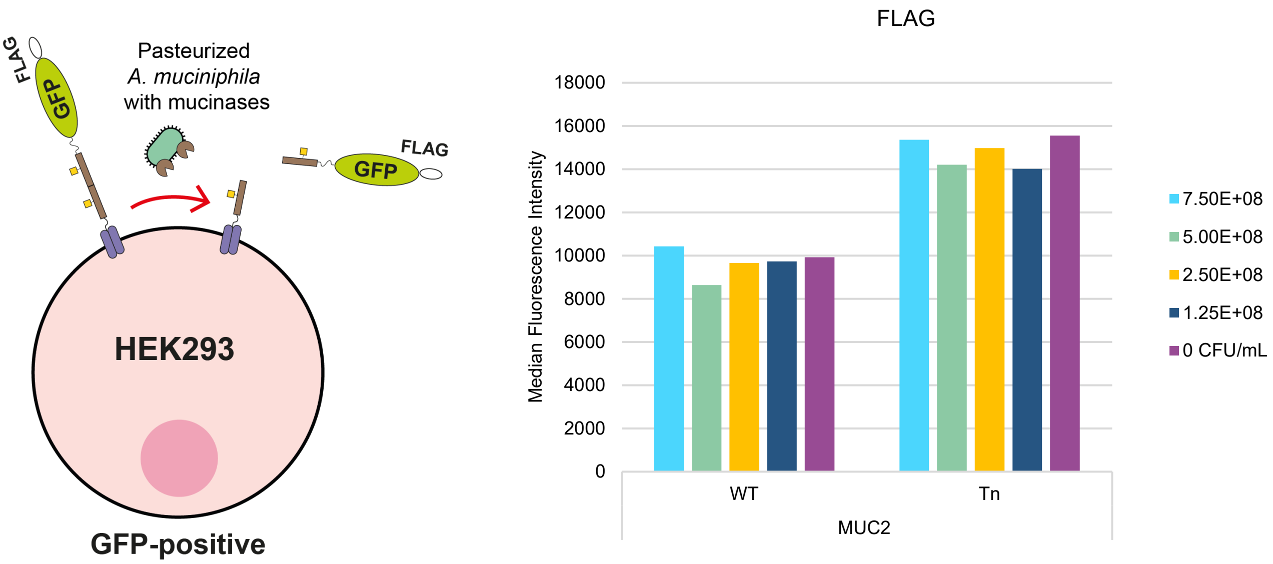

Supplementary Figure 4: Flow cytometry analysis of mucinase activity of pasteurized *A. muciniphila* Schematic representation of assay to test remaining mucinase activity of pasteurized *A. muciniphila* (left), in which HEK293 cells (WT, Tn) transiently expressing the MUC2 TR reporter on the cell surface were incubated with pasteurized *A. muciniphila* (0-7.5 · 10^8 CFU/mL) for 1 hr at 4 °C followed by probing with anti-FLAG antibody (right). Bars represent the median of 10.000 events of one biological replicate. Source data are provided as a Source Data file.

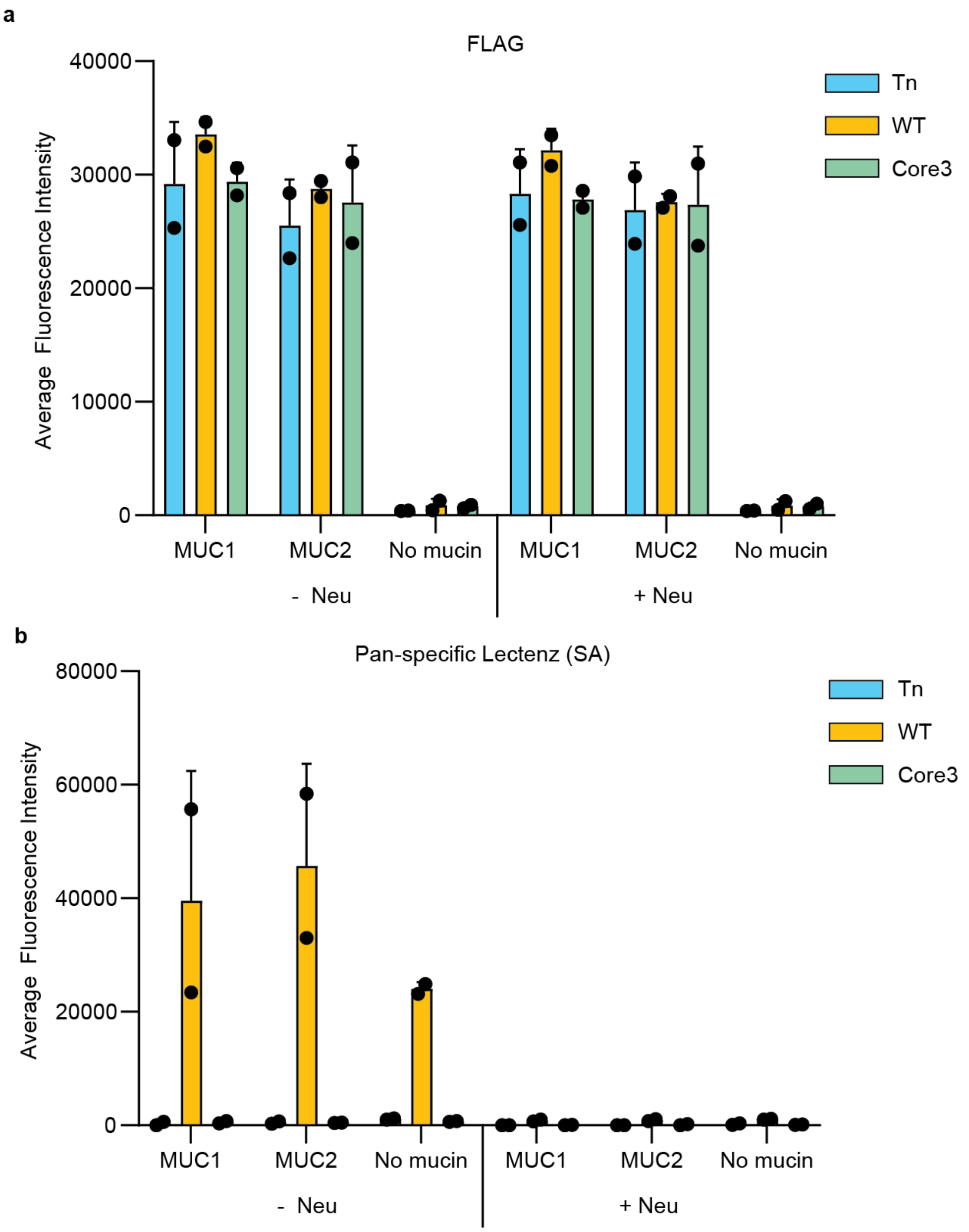

Supplementary Figure 5: Flow cytometry analysis of expression of mucin TR reporters in glycoengineered HEK293 cells. **a** Transient expression of the MUC1 and MUC2 reporters in HEK293 cells detected by an anti-FLAG antibody and **b** the sialic acid pan-specific Lectenz. Cells were tested with and without pretreatment with *C. perfringens* sialidase (10 mU, 1 h). Data points represent the average median fluorescence intensity (MFI) of two biological replicates. Source data are provided as a Source Data file.

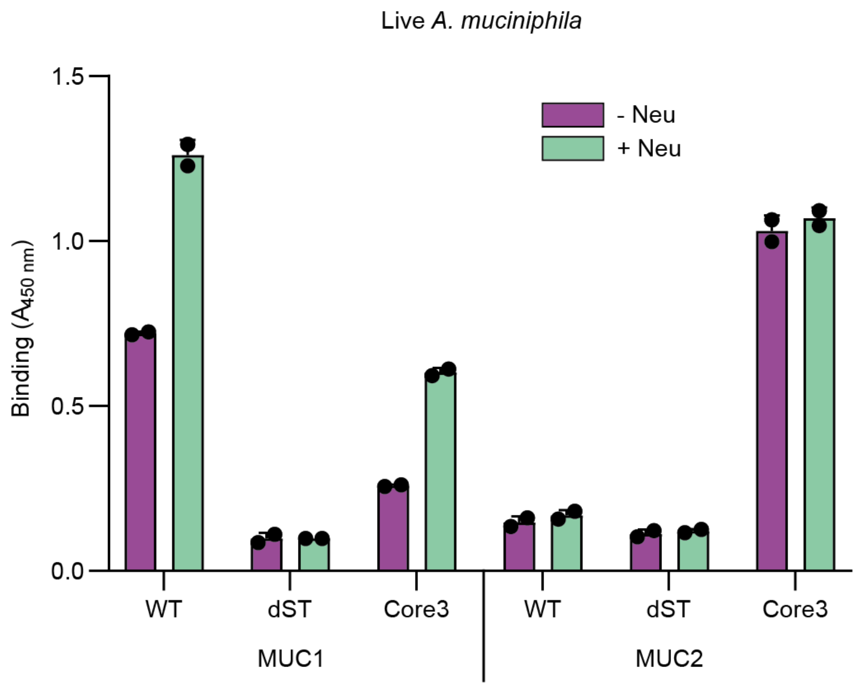

Supplementary Figure 6: ELISA binding assays of live *Akkermansia muciniphila* to mucin TR reporters. Binding of live *A. muciniphila* (1 x 10^9 CFU/mL) to 1 µg/mL mucin TRs (WT, dST and Core3), with and without pretreatment with *Clostridium perfringens* neuraminidase (20 mU overnight). Bars represent the mean ± SD of two technical replicates. Note that absorbance values are lowered compared to **Figure 2**, as TMB incubation time was shorter. Source data are provided as a Source Data file.

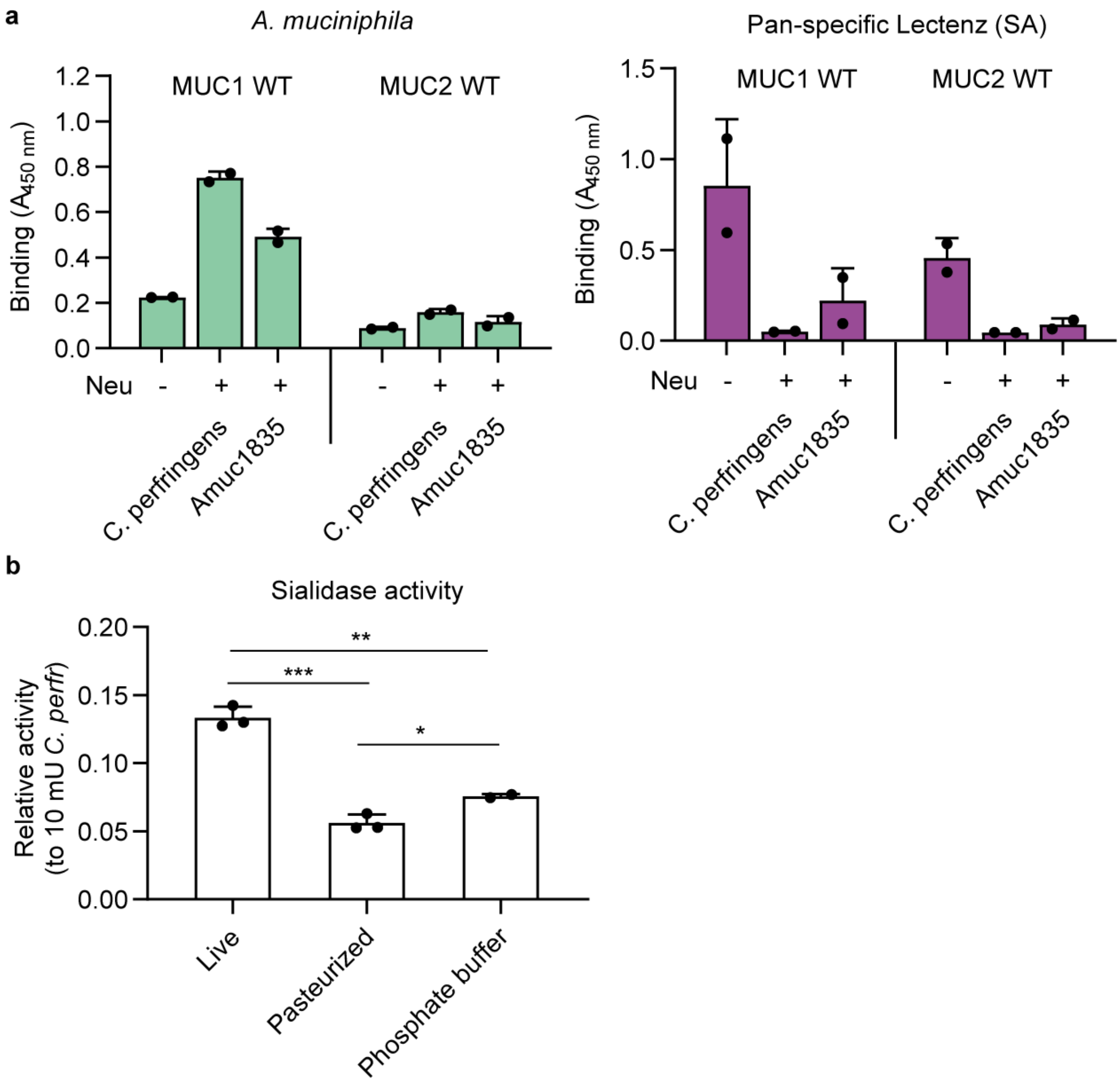

Supplementary Figure 7: Endogenous neuraminidase activity of *A. muciniphila*. a Immobilized recombinant purified mucin reporters (250 ng/mL) were incubated with recombinant *A. muciniphila* Amuc_1835 neuraminidase (0.5 mU) or control *C. perfringens* neuraminidase (2.5 mU) and binding of pasteurized *A. muciniphila* (5 x 10^8 CFU/mL) and the sialic acid binding pan-specific Lectenz (2.0 µg/mL) were tested. Plates were read at 450 nm. Bars represent the mean ± SD of 2 biological replicates. **b** Relative activity of live and pasteurized *A. muciniphila* (5 · 10^8 CFU/mL) compared to 10 mU *C. perfringens* (= 1) for 1 hour at pH 6.0 at 37 °C under oxic conditions. Bars represent the mean ± SD of 3 biological replicates (except phosphate buffer (n = 2)). Residual activity in negative control (phosphate buffer) probably represents background signal of assay. * $p < 0.05$, ** $p < 0.01$, *** $p < 0.001$. Source data are provided as a Source Data file.

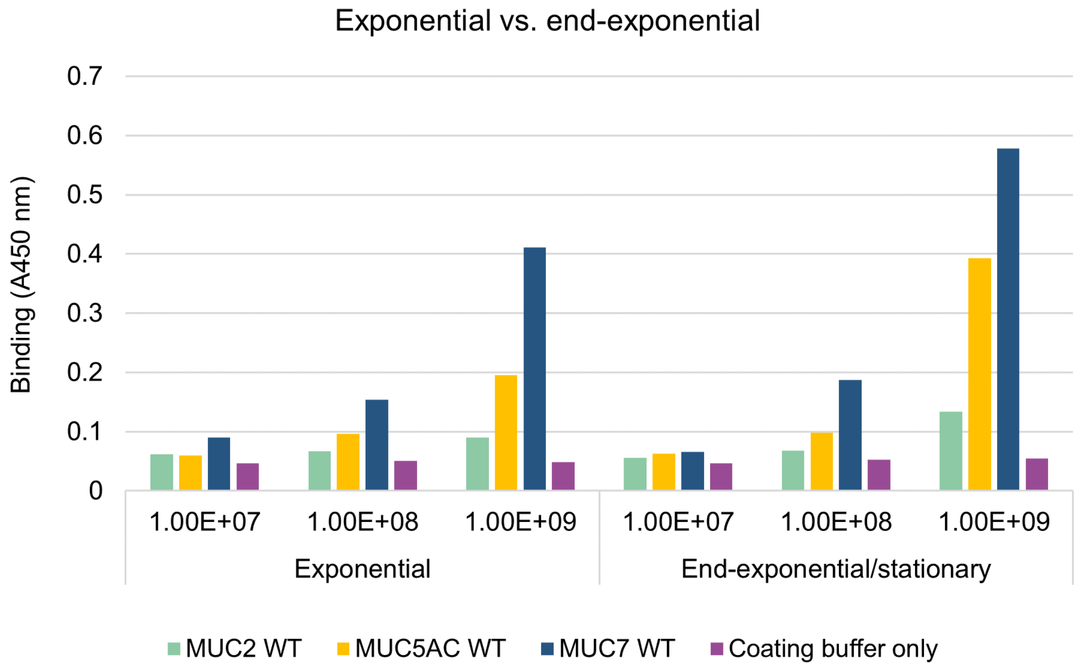

Supplementary Figure 8: Comparison of binding between growth phases. ELISA binding of live *Akkermansia muciniphila* (1 x $10^{7\text{-}9}$ CFU/mL in PLI-P) to mucin TRs (200 ng/mL) at different growth phases. Binding was performed under oxic conditions at 4 °C. Bars represent one technical/biological replicate. Source data are provided as a Source Data file.

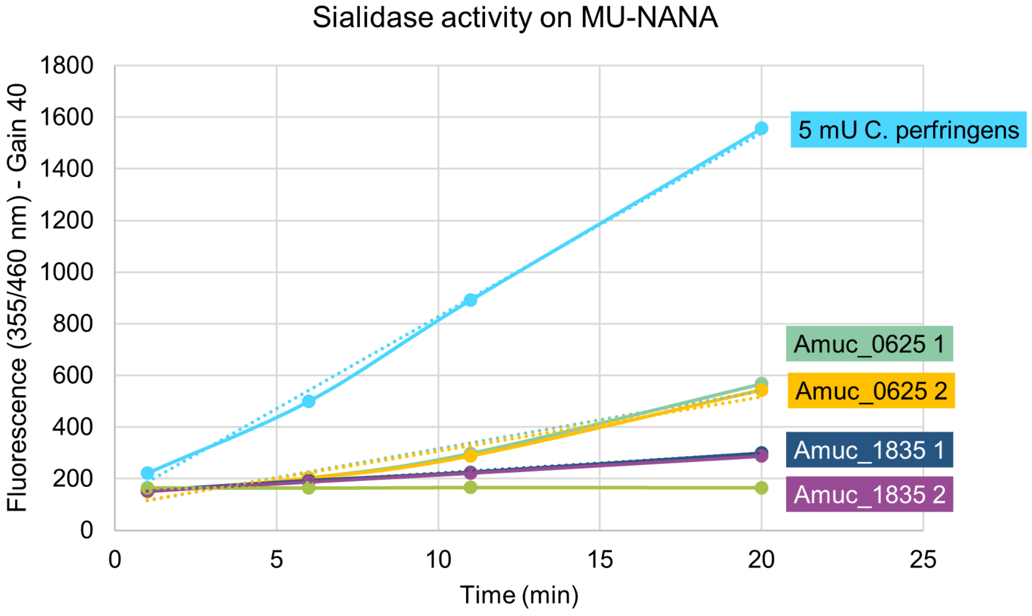

Supplementary Figure 9: Quantification of activity of *C. perfringens* and *A. muciniphila* sialidases using MU-NANA assay. Released 4-methylumbelliferone (MU) after incubation of 5 mU *C. perfringens* or two different aliquots of heterologously expressed Amuc_0625 and 1835 with 1 mM MU-NANA (2′-(4-Methylumbelliferyl)-α-D-N-acetylneuraminic acid). All experiments were performed at pH 6.0 at 37 °C. Stocks of Amuc_0625 and 1835 were diluted 400x with 1 mM MU-NANA in a final reaction of 100 µL. Neuraminidase from *C. perfringens* was bought commercially and diluted to a final concentration of 5 mU. Data points represent one biological replicate. Using a standard curve of MU-NANA the (average) slope of the curves shown are the following: *C. perfringens* (71.3 FI= 0.004 units (µMole MU/min) = 4.5 mU in reaction (in 100 µL)); Amuc_0625 (21.8 ± 0.9 (FI/min) = 0.001 ± 5.8E-05 (µMole MU/min) = 1.4 ± 5.8E-02mU in reaction (in 100 µL), so 5.5 ± 0.2 U/mL stock); Amuc_1835 (7.4 ± 0.4 (FI/min) = 0.0005 ± 2.5E-05 (µMole MU/min) = 0.5 ± 2.5E-02mU in reaction (in 100 µL), so 1.9 ± 0.1 U/mL stock). Source data are provided as a Source Data file.

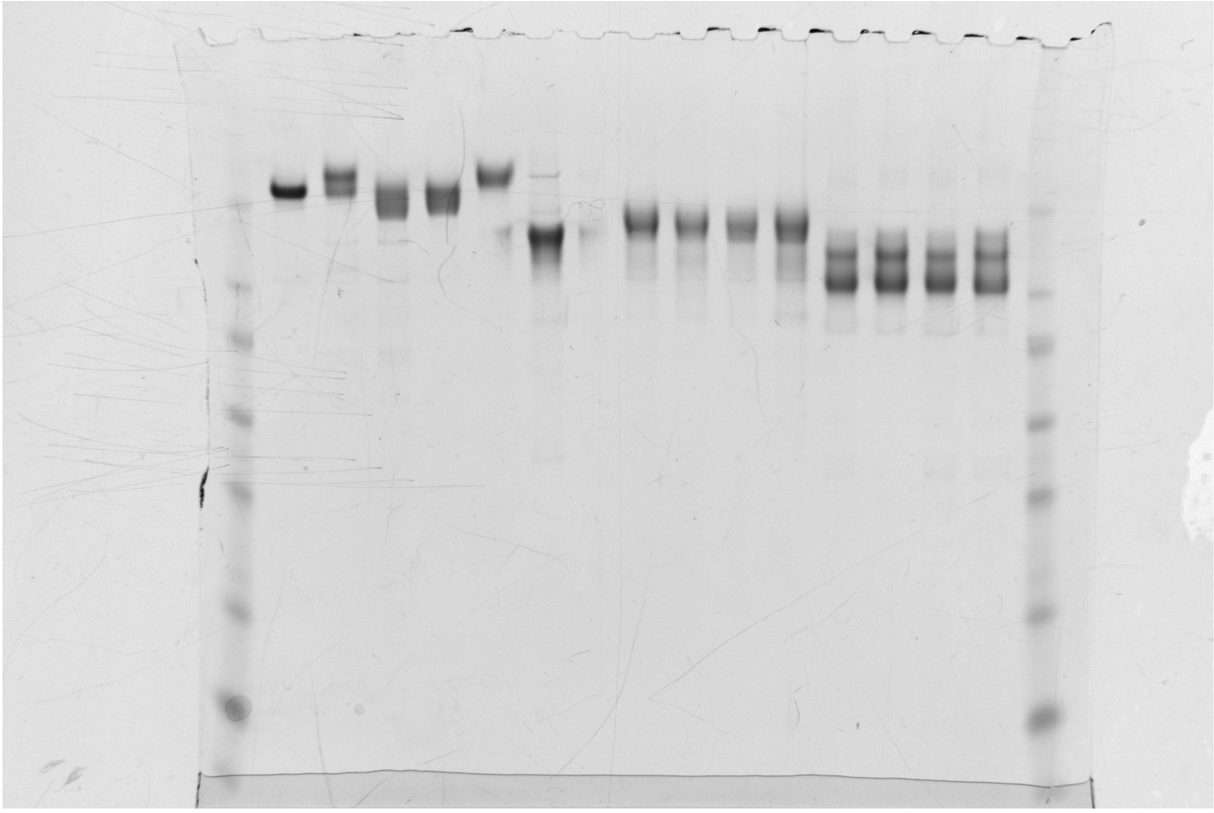

Uncropped scan of gel presented in Supplementary Figure S2. Note: the first six lanes (incl. protein ladder) have been used for Figure S2. The other lanes were used for another experiment, including a N-glycan reporter control (lane 6) and different batches of purified MUC1 (lane 8-16).
