# Supplementary material for: Binding of Akkermansia muciniphila to mucin is O-glycan specific
Source: Nat Commun. 2024 May 29;15:4582. doi: 10.1038/s41467-024-48770-8 (PMC11137150; doi:10.1038/s41467-024-48770-8)
Supplement: Supplementary file 3 — Reporting Summary [file 41467_2024_48770_MOESM3_ESM.pdf]

nature portfolio

Corresponding author(s): Janneke Elzinga
Hanne Tytgat

Last updated by author(s): Apr 29, 2024

Reporting Summary

Nature Portfolio wishes to improve the reproducibility of the work that we publish. This form provides structure for consistency and transparency in reporting. For further information on Nature Portfolio policies, see our Editorial Policies and the Editorial Policy Checklist.

Statistics

For all statistical analyses, confirm that the following items are present in the figure legend, table legend, main text, or Methods section.

n/a	Confirmed	
☐	☒	The exact sample size (*n*) for each experimental group/condition, given as a discrete number and unit of measurement
☐	☒	A statement on whether measurements were taken from distinct samples or whether the same sample was measured repeatedly
☐	☒	The statistical test(s) used AND whether they are one- or two-sided<br>*Only common tests should be described solely by name; describe more complex techniques in the Methods section.*
☒	☐	A description of all covariates tested
☐	☒	A description of any assumptions or corrections, such as tests of normality and adjustment for multiple comparisons
☐	☒	A full description of the statistical parameters including central tendency (e.g. means) or other basic estimates (e.g. regression coefficient) AND variation (e.g. standard deviation) or associated estimates of uncertainty (e.g. confidence intervals)
☐	☒	For null hypothesis testing, the test statistic (e.g. *F*, *t*, *r*) with confidence intervals, effect sizes, degrees of freedom and *P* value noted<br>*Give P values as exact values whenever suitable.*
☒	☐	For Bayesian analysis, information on the choice of priors and Markov chain Monte Carlo settings
☒	☐	For hierarchical and complex designs, identification of the appropriate level for tests and full reporting of outcomes
☒	☐	Estimates of effect sizes (e.g. Cohen's *d*, Pearson's *r*), indicating how they were calculated

Our web collection on statistics for biologists contains articles on many of the points above.

Software and code

Policy information about availability of computer code

Data collection	Gen5 software, Sony SA3800 software
Data analysis	FlowJo LLC v. 10, Microsoft Excel, Graphpad Prism 10, Thermo Proteome Discoverer 2.2.0.388, GlycoWorkbench 2.1 (build 146), the Thermo Xcalibur qual browser 3.0.63

For manuscripts utilizing custom algorithms or software that are central to the research but not yet described in published literature, software must be made available to editors and reviewers. We strongly encourage code deposition in a community repository (e.g. GitHub). See the Nature Portfolio guidelines for submitting code & software for further information.

Data

Policy information about availability of data

All manuscripts must include a data availability statement. This statement should provide the following information, where applicable:

- Accession codes, unique identifiers, or web links for publicly available datasets
- A description of any restrictions on data availability
- For clinical datasets or third party data, please ensure that the statement adheres to our policy

All elements necessary to allow interpretation and replication of results, are provided in the Supplementary Information. The mass spectrometry proteomics data have been deposited to the ProteomeXchange Consortium via the PRIDE repository with the dataset identifier PXD051738. Source data are provided with this paper in the Source Data file.

Research involving human participants, their data, or biological material

Policy information about studies with human participants or human data. See also policy information about sex, gender (identity/presentation), and sexual orientation and race, ethnicity and racism.

Reporting on sex and gender	NA
Reporting on race, ethnicity, or other socially relevant groupings	NA
Population characteristics	NA
Recruitment	NA
Ethics oversight	NA

Note that full information on the approval of the study protocol must also be provided in the manuscript.

Field-specific reporting

Please select the one below that is the best fit for your research. If you are not sure, read the appropriate sections before making your selection.

☒ Life sciences ☐ Behavioural & social sciences ☐ Ecological, evolutionary & environmental sciences

For a reference copy of the document with all sections, see nature.com/documents/nr-reporting-summary-flat.pdf

Life sciences study design

All studies must disclose on these points even when the disclosure is negative.

Sample size	No sample size calculations were done. Sample size was chosen based on experience of the investigators with similar experiments conducted multiple times and previously published. See also "Replication"
Data exclusions	No data were excluded from the analyses.
Replication	All attempts to repeat the experiment were successful and the inclusion of multiple concentrations of A. muciniphila or mucins were tested within one experiment to support the data. All ELISA experiments shown in the main figures were repeated at least 3 times under the same conditions (3 replicates averaged) or under slightly different experimental conditions with similar trends (one representative experiment shown). For ELISA with Amuc0625 and Amuc1835, only two biological replicates were performed because of limited availability of enzymes. Flow cytometry experiments in the main figures were performed 2 times.
Randomization	The experiments were not randomized, as randomization is irrelevant to our study.
Blinding	The investigators were not blinded to allocation during experiments and outcome assessment. Bllinding is irrelevant to our study since there is no selection bias during the experiments or data collection

Reporting for specific materials, systems and methods

We require information from authors about some types of materials, experimental systems and methods used in many studies. Here, indicate whether each material, system or method listed is relevant to your study. If you are not sure if a list item applies to your research, read the appropriate section before selecting a response.

Materials & experimental systems

n/a	Involved in the study	
☐	☒	Antibodies
☐	☒	Eukaryotic cell lines
☒	☐	Palaeontology and archaeology
☒	☐	Animals and other organisms
☒	☐	Clinical data
☒	☐	Dual use research of concern
☒	☐	Plants

Methods

n/a	Involved in the study	
☒	☐	ChIP-seq
☐	☒	Flow cytometry
☒	☐	MRI-based neuroimaging

Antibodies

Antibodies used	Rabbit anti-A. muciniphila serum (kind gift of Dr. J. Reunanen (University of Helsinki))<br>HRP-conjugated goat anti-rabbit IgG (H+L) (Invitrogen, Cat: A16096)<br>anti-FLAG M2-Peroxidase-HRP-conjugated MAb (Sigma, Cat: A8592, Clone M2)<br>APC-conjugated anti-FLAG (Biolegend, Cat: 637308, Clone: L5)<br>Goat anti-Rabbit IgG, Alexa Flour 647 (Invitrogen by Thermo Fisher, Cat: A21245)
Validation	Commercially purchased antibodies directed against standard protein tags were validated on cell lines transfected with proteins with or without the relevant tag.<br>APC-conjugated anti FLAG (Biolegend), as stated on the website: Each lot of this antibody is quality control tested by intracellular immunofluorescent staining with flow cytometric analysis.<br>Anti-Akkermansia serum is validated in Reunanen et al. (2015) in AEM (http://dx.doi.org/10.1128/AEM.04050-14)

Eukaryotic cell lines

Policy information about cell lines and Sex and Gender in Research

Cell line source(s)	HEK293 cells were obtained from ATCC (CRL-1573)<br>Glycoengineered HEK293 cell lines are available as part of the cell-based glycan array resource and published in Nason et.al., Nat Commun 2021 and Narimatsu et.al., Mol Cell 2019.
Authentication	No specific authentication of cell lines used apart from separate handling of original obtained vials throughout entire project. Each individual engineered HEK293 clones were confirmed multiple times by HEK293 gene specific IDAA and Sanger sequencing in the target gene area(s).
Mycoplasma contamination	All cell lines tested negative for mycoplasma contamination
Commonly misidentified lines<br>(See ICLAC register)	None of the cell lines used are listed in the ICLAC database.

Plants

Seed stocks	*Report on the source of all seed stocks or other plant material used. If applicable, state the seed stock centre and catalogue number. If plant specimens were collected from the field, describe the collection location, date and sampling procedures.*
Novel plant genotypes	*Describe the methods by which all novel plant genotypes were produced. This includes those generated by transgenic approaches, gene editing, chemical/radiation-based mutagenesis and hybridization. For transgenic lines, describe the transformation method, the number of independent lines analyzed and the generation upon which experiments were performed. For gene-edited lines, describe the editor used, the endogenous sequence targeted for editing, the targeting guide RNA sequence (if applicable) and how the editor was applied.*
Authentication	*Describe any authentication procedures for each seed stock used or novel genotype generated. Describe any experiments used to assess the effect of a mutation and, where applicable, how potential secondary effects (e.g. second site T-DNA insertions, mosiacism, off-target gene editing) were examined.*

Flow Cytometry

Plots

Confirm that:

- [x] The axis labels state the marker and fluorochrome used (e.g. CD4-FITC).
- [x] The axis scales are clearly visible. Include numbers along axes only for bottom left plot of group (a 'group' is an analysis of identical markers).
- [x] All plots are contour plots with outliers or pseudocolor plots.
- [x] A numerical value for number of cells or percentage (with statistics) is provided.

Methodology

Sample preparation	HEK293 cells were suspended in FACS buffer (PBS plus 1% BSA (w/v)) and incubated with serial dilutions of A. muciniphila on ice or at 4 °C for 1 h, followed by incubation with polyclonal anti-serum to A. muciniphila (1:1000) and cross-absorbed Alexa Fluor™ 647 -conjugated goat anti-rabbit IgG (2 µg/mL, Invitrogen) for 1 h at 4°C before repeated washing and analysis.<br>Checking the desialylation level, cells were incubated with 2.0 µg/mL biotinylated SiaFindTM pan-specific Lectenz® (Lectenz Bio) pre-incubated with 2 µg/mL Alexa Fluor™ 647-conjugated streptavidin for 1 h at 4°C before repeated washing and analysis.
Instrument	SONY SA3800 Spectral cell analyzer

Software	FlowJo Version 10 was used
Cell population abundance	Not applicable. Gating was performed only to exclude dead cells and doublets.
Gating strategy	The dead cells were excluded based on forward and side scatter area (FSC-A and SSC-A) parameter. Cells expressing mucin-GFP reporter (GFP+) or not expressing mucin-GFP reporter (GFP-) were gated by level of GFP (488 nm).

☒ Tick this box to confirm that a figure exemplifying the gating strategy is provided in the Supplementary Information.
